# The Hospital World

**Published:** 1911-08-05

**Authors:** 


### The Hospital World.

### Death of an Ex-M.P. Practitioner.

It is announced that Mr. Donald Macgregor, M.R.C.S., L.R.C.P., and ex-M.P. for Inverness-shire, has died at Ealing at the age of seventy-two. Mr. Macgregor, who studied at Edinburgh, qualified in 1864, and at one time was medical superintendent of Barnhill Hospital and Asylum, Glasgow. He had retired from practice some years before his death.

### Death of Dr. Bulstrode.

Dr. Herbert Timbrell Bulstrode, M.D., of H.M. Local Government Board, has died in London at the age of fifty-two. He was an M.D. of Cambridge, studied at St. Thomas', and lectured on preventive medicine at Charing Cross Hospital Medical School. It is stated that he visited Aldershot officially last week, and the next morning was found dead. He was distinguished in the two branches of the public health work relating to the investigation of tuberculosis and to the agency of shellfish in the causation of disease, and was the author of many reports on both subjects. The Board published his "Sanatoria for Consumption" as a standard work. His latest report was on the outbreak of plague last autumn among the rats in Essex and Suffolk.

### The Hampton Court Vine.

The London hospitals are shortly to receive their annual grant of grapes from the famous vine at Hampton Court, which has proved a generous benefactor to them for many years. In the old days the vine, which was planted in 1763, used to bear as many as one thousand bunches, but now the number has been reduced artificially to two or three hundred. It is stated these are ready for cropping.

### Death of Dr. Nélaton.

The surgeon Charles Nélaton (a name famous in Parisian surgery) has died suddenly in Paris at the age of sixty. This news has affected a wide circle, for, besides being well known, he was popular alike as a teacher and with his patients. He was the son, it should be remembered, of the great Nélaton, Napoleon III.'s surgeon. He went through his medical course at Paris, qualified as hospital house surgeon in 1876, and obtained his *doctorat en médecine* in 1880 with a still remembered thesis on "The Effusions of Blood in the Pleuræ, following on Trauma." In 1881 Charles Nélaton was elected by competitive examination surgeon of the hospitals of Paris. He did not cease dividing his exertions between his hospital duties and his personal researches, which enabled him to publish successively his original works: "Le Tubercule dans les affections Chirurgicales," "Le Rapport du Traumatisme avec les affections Cardiaques," "Les Causes de l'irréductibilité des Luxations Anciennes de la Hanche." He was elected agrégé of the Faculty of Paris in 1889. The Boucicault Hospital, of which he was surgeon, loses a friend and protector. It is interesting to add that the French papers announce among the new chevaliers of the *Légion d'honneur* Dr. Broca, surgeon of the Hospital for Sick Children. "M. Caillaux, President of the Council," says the *Figaro*, "had likewise proposed Dr. Nélaton for a Chevalier's Cross, but the illustrious practitioner has just died. Dr. Nélaton, whose modesty equalled his scientific knowledge, had requested the President of the Council to bestow the Cross intended for him on one of his friends, a doctor like himself, whose services, he said, were much more important than his own. But M. Caillaux had resisted these solicitations, and Dr. Nélaton has just died at the moment when this distinction would have been conferred on him. Now, perhaps, the President of the Council will remember the friend whom Dr. Nélaton recommended to him so warmly."

### Dr. John Beddoe: Crimean Veteran.

Dr. John Beddoe, M.D., F.R.C.P., F.R.S., the anthropologist, has died at Bradford-on-Avon, aged eighty-four. Dr. Beddoe was born in 1826, and studied at University College, London, Edinburgh University, and Vienna, qualifying as M.D. of Edinburgh in 1853, and F.R.C.P. twenty years later. He served in the Civil Hospital Staff in the Crimean War, and practised at Clifton from 1857 to 1891. He was an inveterate frequenter and office-holder of medical and non-medical societies, and his quondam appointments were those of physician to the Bristol Royal Infirmary, and resident physician to the Edinburgh Royal Infirmary. His contributions to anthropological science included "Contribution to Scottish Ethnology," "Stature and Bulk of Man," and "The Races of Britain."

### The Women's Hospital, Edinburgh.

The recently completed wing of the Edinburgh Hospital and Dispensary for Women and Children at Whitehouse Loan, Edinburgh, which the Queen opened last week, belongs to an institution with a rather curious history. It was established in 1878 by Dr. Sophia Jex-Blake, who started a women's dispensary in Grove Street, which was transformed in 1885 into a cottage hospital. In 1896 the site of the buildings was purchased, and till the present extension was opened the institution's accommodation amounted to sixteen beds and two cots. To-day that number has been increased to forty. One of the beds in the new wards has been endowed by Lady Houldsworth. The Queen was received by Miss S. E. S. Mair, chairman of the Executive Committee, Mr. Balfour Paul, the architect of the new wing, Miss Evelyn MacLaren, treasurer, and Mrs. James C. Johnston, honorary secretary. It must not be forgotten that the Royal Hospital for Sick Children in Sciennes Road was on the route to Holyrood, and a halt was made at the Longmore Hospital, where Mr. Walter Macgregor, secretary and treasurer, together with a section of the staff, was in attendance. Dr. Affleck recalled the fact that the Queen had visited Longmore in 1893.

### Nelson and his Hospital.

At the laying of the foundation-stone of the new Nelson Hospital for South Wimbledon and Merton by the Duchess of Sutherland, Alderman Summerhays recalled that the hospital was the outcome of the South Wimbledon District Hospital formed eleven years ago. The committee had received a special grant in recognition of the work accomplished, and the new hospital had the special sympathy of the present Lord Nelson, who was not only a resident of Merton, but one who greatly loved it. They had therefore decided that the name of Nelson should be associated with the new institution. They had set out to put up a hospital that would contain twenty-four patients, and they were providing in adjoining ground sufficient space to make the hospital, one of these days when the need arose, a building for forty patients. Colonel Mitchell said that for many years they had felt the need of a hospital for South Wimbledon and Merton, where there was a vast amount of distress. The Duchess of Sutherland was presented with a trowel and mallet in a box of "Victory" oak, and in reply she said she would cherish them in remembrance of her old-time hero Lord Nelson, some of whose letters to her ancestor Sir James Sinclair Erskine, in the time of the Peninsular War, she had still. As The Hospital stated on July 1 (page 341), it is believed that Nelson started from Merton for Trafalgar. Merton is also interesting as the place chosen by William Morris for his workshop, and the famous blue-dye vats are there still.

